# 191 Process of Care and Hand Hygiene in Long-term Care Facilities (LTCF)

**DOI:** 10.1017/ash.2026.10581

**Published:** 2026-06-23

**Authors:** Armaghan-e-Rehman Mansoor, Joseph Hurt-Mueller, Wael Ibrahim, Amy Gewirtz, Joel Howard, Randal De Souza, Danielle Casaus, Katie Wallace, Derek Forster

**Affiliations:** 1 University of Kentucky; 2 University of Kentucky Healthcare; 3 University of Kentucky, College of Medicine; 4 University of Kentucky/UK HealthCare; 5 Golisano Children’s at UK; 6 University of Kentucky School of Medicine

## Abstract

**Background:** Multiplex respiratory panels are commonly utilized in the clinical care of respiratory infections. This usage occurs despite their high cost and absence of impact on clinical decision making. Guidance from national societies such as the Society of Hospital Medicine and American Association of Family Practice suggest limiting use to immunocompromised hosts or targeted testing based on seasonal prevalence, however clinical practice often does not mirror these recommendations. **Methods:** We conducted a retrospective review of the ePlex repiratory panel, an automated polymerase chain reaction for detection and identification of multiple respiratory viral and bacterial nucleic acids, at University of Kentucky Healthcare during July 2024-June 2025. Test volumes were stratified by location and month. We assessed the frequency of insurance denials, and the estimated cost of unreimbursed testing. These results were then utilized to create a multi-pronged stewardship intervention. **Results:** A total of 15,320 tests were ordered, with 32.2% reporting at least one positive analyte, most commonly rhinovirus/enterovirus (25.5%) (Figure 1). Tests were most frequently ordered inpatient (7315 tests, 47.7%). Of the 3787 (24.7%) outpatient tests, urgent and primary care settings accounting for the largest propotion (46.5%). Insurance denials led to an unreimbursed cost of 1.78 million USD. Testing peaked in the winter months (1873 tests in December 2024), with a nadir of 935 tests in August 2024 (Figure 2). The intervention comprised three domains. First, the intervention was discussed and approval sought from key stakeholders including Lab formulary committee, inpatient and outpatient medical directors, Infectious Diseases, Laboratory services, and Pharmacy. Second, a comprehensive modification of the EMR including creation of nested order panels that led ordering users through clinical scenarios to determine if testing was indicated, selection of an appropriate use criteria if the test was ordered, and redirecting keywords to the order panel instead of the individual test order. Last, a patient/caregiver handout was created on supportive care for viral infections, and the lack of impact of multiplex testing in non-immunocompromised hosts. After the interventions went live in late-November 2025, test volume saw a significant decline with a 29% decline in November 2025, and a 73% decline in December 2025 (Figure 2). **Conclusion:** Our preliminary results show remarkable impact of a comprehensive diagnostic stewardship effort targeting appropriate test ordering of a multiplex viral respiratory panel. We plan to monitor long-term test usage, as well as impact on unreimbursed costs.

